# Vocal Phenology of Forest Tinamous Along a Latitudinal Gradient: Effects of Daylength and Precipitation

**DOI:** 10.1002/ece3.73741

**Published:** 2026-06-12

**Authors:** Oscar Laverde‐R., Santiago Muñoz Bolaños, Gustavo A. Londoño, Christian Devenish

**Affiliations:** ^1^ Departamento de Biología, Facultad de Ciencias Pontificia Universidad Javeriana Bogotá Colombia; ^2^ Facultad de Ciencias Naturales Universidad ICESI Cali Valle del Cauca Colombia; ^3^ School of Life Sciences Keele University Staffordshire UK

**Keywords:** citizen science, eBird, seasonality, singing patterns, Tinamidae, tropical region

## Abstract

The phenology of bird vocalizations is increasingly employed to understand avian reproduction and territoriality, among other ecological aspects. Although precipitation is ostensibly the major driver of seasonality at the equator, subtle photoperiodic signals are discernible in daylength, with a typical variation of 20 to 25 min over the entire year. However, in hyper‐diverse areas, such as the tropics, environmental cues for phenological patterns are relatively small compared to temperate regions, requiring long‐term data to identify patterns of vocal seasonality. One such source of data comes from the increasing numbers of bird visual and aural records generated by citizen science initiatives, easily accessible through online platforms such as eBird. First, we validated the use of eBird citizen science data for vocal phenology research, demonstrating strong concordance with independent audio recordings. Then, we assessed the vocal phenology of forest tinamous using a large eBird dataset. This work tested the hypotheses that the vocal phenology of tropical forest tinamous is influenced by changes in daylength and precipitation. To test the effect of changes in daylength and precipitation, we grouped records of forest tinamous in five regions with similar precipitation regimes over four latitudinal bands. After filtering, we obtained a total of 44,071 records of 16 forest tinamou species between January 2000 and December 2022. We found that vocal phenology in tropical forest tinamous can be predicted by changes in precipitation but is primarily influenced by changes in daylength. There appears to be a clear difference in singing patterns between the broad regions defined by precipitation regimes. In wetter regions, the effect of precipitation was stronger. Forest tinamous vocalize mainly when precipitation is low, especially in humid regions with similar climatic regimes. They also tend to sing more when days become either longer or shorter, with the lowest vocal activity at 12 h of daylength. Questions such as these, concerning tropical phenology, have been elucidated through the contribution of citizen science to this burgeoning field.

## Graphical abstract

Based on an analysis of eBird records, this study finds that the vocal patterns of tropical forest tinamous are primarily influenced by changes in daylength, with a secondary effect of precipitation. Birds sang more when days were longer or shorter than 12 h and during drier periods, especially in wetter regions. The research demonstrates the value of long‐term citizen science data in uncovering the vocal phenology of birds in hyper‐diverse, aseasonal tropical environments.

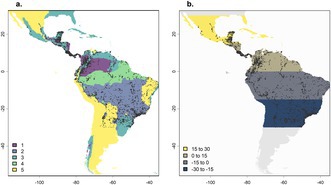

## 1 Introduction

Phenology is the study of recurrent biological events, often tied to seasonal and interannual variations in climatic cycles, such as flowering or fruiting in plants and singing or breeding in birds (Abernethy et al. 2018; Berman et al. 2023). In the last decade, phenology has increasingly gained the attention of the global scientific community due to shifts in phenological events associated with climate change (Berman et al. 2023; Gutiérrez‐Carrillo et al. 2024). However, to date, this interest has mostly focused on temperate regions, particularly in relation to seasonal migration patterns (Scheffers et al. 2016; Gutiérrez‐Carrillo et al. 2024). Although the tropics do not show marked seasons as in temperate regions, variations in precipitation and daylength appear to be important triggers for flowering in plants (Borchert et al. 2005) and reproduction in birds (Buxton et al. 2016; Hau et al. 1998; Oppel et al. 2013; Renthlei et al. 2022; Stutchbury and Morton 2022; Wikelski et al. 2000). In temperate zones, large seasonal shifts in daylength provide a reliable, long‐term predictive cue for reproduction. In the tropics, however, the predictive value of small annual photoperiodic changes is unclear.

Close to the equator, seasonality is determined mostly by precipitation (Boyle et al. 2020). In most tropical areas, two seasons can be defined, a dry season and a wet season. Primary productivity in tropical regions also tends to be seasonal (Cleveland et al. 2011) due to its relationship with the availability of nutrients such as carbon, nitrogen, and phosphorus, which follow recirculation cycles and tend to increase rapidly with increased precipitation after dry periods (Sun and Du 2017).

These changes in climate, particularly precipitation and forest productivity—and, consequently, insect abundance—are important for bird reproduction and vocal behavior. For instance, in the Red‐throated Ant‐tanager ( *Habia fuscicauda* ), a socially monogamous species, males predominantly sing during the breeding season, peaking just before the rains arrive (Chiver et al. 2015). Similarly, the drumming behavior of the Pileated Woodpecker ( *Dryocopus pileatus* ) peaks in mid‐March, prior to the onset of breeding activities before the rainy season (Tremain et al. 2008).

Bird vocalizations mediate most intra‐ and interspecific interactions, including territory establishment (De Kort et al. 2009), mate defense (Adachi and Soma 2019), courtship (Nowicki et al. 2002), and flock organization (Gayk and Mennill 2023). In the tropics, two vocal phenology strategies have been recognized: seasonal vocalizations, often linked to reproduction (Chiver et al. 2015; Demko and Mennill 2018; Vokurková et al. 2018), and non‐seasonal (Stutchbury and Morton 2022). Taxa with seasonal vocalizations sing only at certain times of the year, mainly during the dry season just before the beginning of the rainy season when reproduction peaks (Vokurková et al. 2018). These groups include tinamous (Negret et al. 2015; Pérez‐Granados et al. 2020), curassows (Baldo and Mennill 2011), blackbirds (Stutchbury et al. 1998), some tanagers (Chiver et al. 2014; Quispe et al. 2017), warblers (Demko and Mennill 2018), and brushfinches (Avendaño 2021). In contrast, avian species that tend to occupy small and permanent territories, such as antbirds (Wikelski et al. 2000) and wrens (Topp and Mennill 2008), sing throughout the year. Nevertheless, the overall vocal activity of most Neotropical species broadly increases with changes in precipitation and daylength, generating a seasonality in their collective vocalization intensity (Agostino et al. 2020; Pérez‐Granados and Schuchmann 2023; Topp and Mennill 2008; Quispe et al. 2017; Vokurková et al. 2018; Wikelski et al. 2000). Other factors influencing seasonal vocal activity include lunar cycles (Braga and Motta‐Junior 2009).

Birds of the family Tinamidae are terrestrial, crepuscular species with limited flight capacity, generalist feeding habits, and cryptic coloration. Additionally, they have very distinctive vocalizations among species, facilitating identification without visual detection (Brennan 2004). Tinamous tend to produce a limited number of vocalizations, generally referred to as calls, but that do follow seasonal patterns (Pérez‐Granados and Schuchmann 2021; Skutch 1963), suggesting a seasonal breeding period associated with peaks in vocal activity. In the tropics, seasonal variation in vocal phenology of Tinamidae has been linked to several factors, such as daylength (Lancaster 1964), lunar cycles (Pérez‐Granados and Schuchmann 2021), and climate (e.g., rainfall; Pérez‐Granados and Schuchmann 2020).

To test the effect of rainfall and daylength on vocal phenology, we explored vocalization dynamics in tinamous throughout their Neotropical distribution using a 22‐year dataset of eBird records. eBird is a citizen science platform populated by millions of bird observations from around the world (eBird 2023; ebird.org). The information collected by this platform has been widely used to explain patterns of spatial distribution and species migration (Walker and Taylor 2017). Specifically, we asked whether the number of tinamou vocalizations per month is related to mean monthly precipitation and/or daylength and whether any relationships vary in strength by latitude (i.e., more marked variation in daylength) or by regional precipitation regimes.

## 2 Materials and Methods

### 2.1 Species Selection

Forest tinamous are the most diverse clade in the family Tinamidae, with three genera (*Nothocercus*, *Crypturellus*, *Tinamus*) and 29 species. They generally inhabit the understorey of forested areas where vegetation density is very high and visibility is very low (Bertelli and Tubaro 2002; Brennan 2004). Because of these cryptic conditions, tinamous are more often heard than seen, making their vocalizations a critical tool for monitoring and phenological studies.

### 2.2 Data Collection

We downloaded all records of the 29 species of tinamous from April 2023 eBird Basic Dataset (eBird 2023). Using the *auk* package (Strimas‐Mackey et al. 2018) implemented in R (R Core Team 2023), we extracted eBird records between January 2000 and December 2022. We acknowledge that semi‐structured citizen science data sets do not provide the same quality or completeness in space and time as systematic survey data (Johnston et al. 2021; Freeman et al. 2022; Rueda‐Uribe et al. 2024), yet this is the largest dataset available at the continental level for this cryptic and shy neotropical family.

**FIGURE 1:**
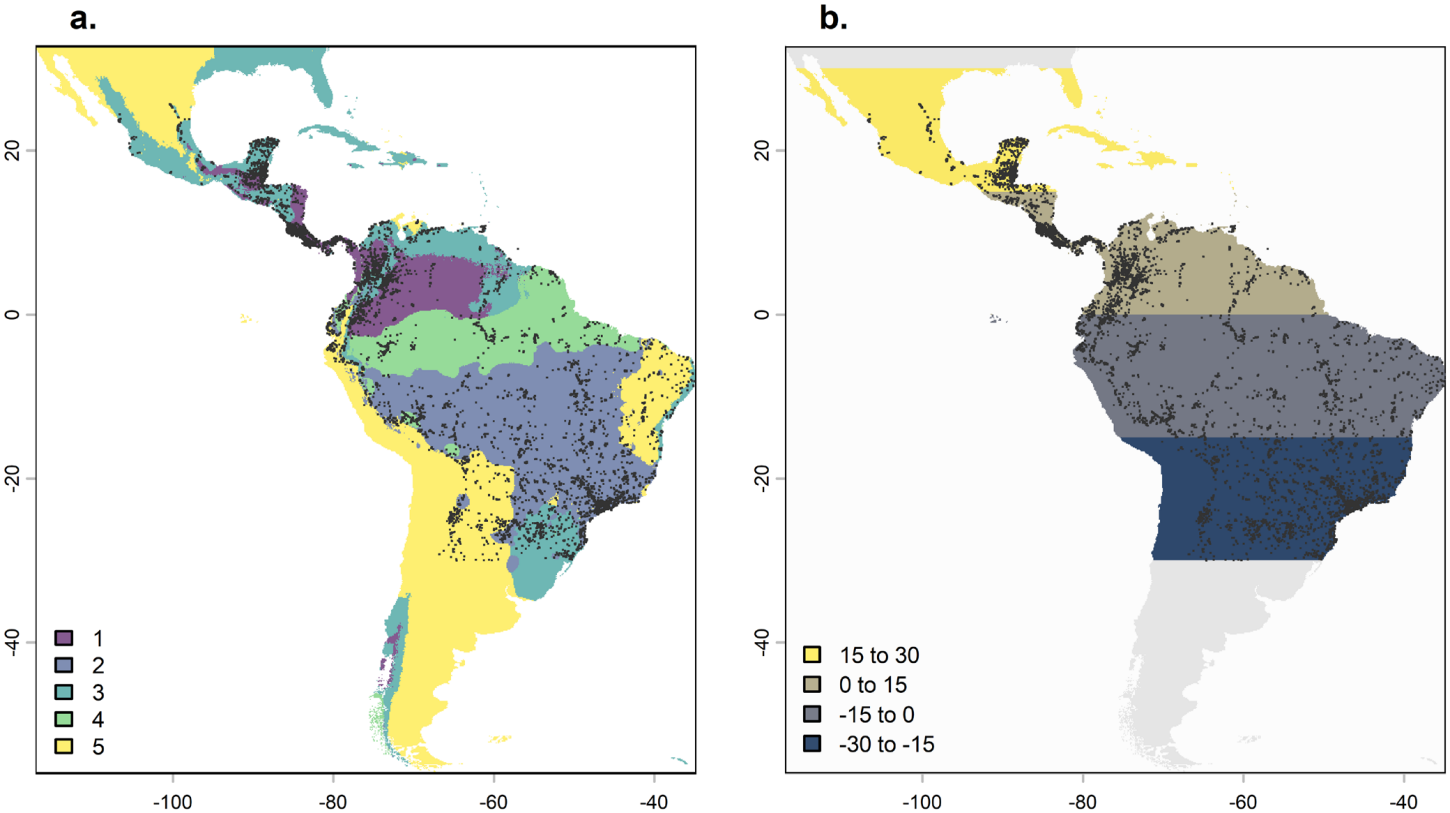
eBird tinamou observations used in this study (black dots), shown with (a) five precipitation regimes and (b) four latitudinal bands. See below for illustrative plots of yearly daylength per latitudinal band (Figure 4a–d) and precipitation regimes (Figure 5a–e).

Further, we have implemented several quality filters to improve data (Johnston et al. 2021), as follows. Given that a single coordinate location is provided for each survey (which may span several kilometers or hours), we minimized location inaccuracy by filtering survey effort to between 0 and 5 km in distance, and less than 60 min in duration. To further improve quality of records, we restricted data sets to those with less than 15 observers within the group, and where the count of individuals recorded was less than five across the whole survey. We only used observations that had been recorded as part of a complete survey, that is, where the observer claimed to have noted all species seen or heard. Finally, we removed species with less than 300 observations across the whole data set (*n* = 1187), leaving a total of 44,993 observations of 16 species (Figure 1).

### 2.3 Validation of eBird Data

Considering tinamou behavior and cryptic coloration that makes the forest genera difficult to see (Brennan 2004), as well as their ability to produce loud and distinctive vocalizations (Solano‐Ugalde et al. 2018), we assume that the vast majority of eBird records correspond to vocalizations. We corroborated this assumption using two approaches: we compared the eBird data to two independent audio data sets, and we also evaluated the comments associated with the eBird records.

To compare with independent audio data, we selected two species with narrow latitudinal limits ( *Crypturellus tataupa* and *C. boucardi* ) and compared the seasonal patterns generated from eBird data against those data sourced from two audio repositories (xeno‐canto; xeno‐canto.org and Macaulay Library of Natural Sounds; macaulaylibrary.org). We modeled the annual vocal phenology for each data source using generalized additive models (GAMs) with the *mgcv* package in R (Wood 2017), generating smoothed curves of scaled monthly vocal activity. To objectively assess congruence, we performed cross‐correlation analysis on the fitted curves using the function *ccf* (R Core Team 2023). This method computes the Pearson correlation coefficient across a range of time lags (±6 months) to identify the optimal alignment and the corresponding maximum correlation coefficient, testing whether both platforms captured the same underlying phenological signal.

To evaluate the occurrence record comments, we extracted data from both comments on each observation, where available, and eBird behavior codes (eBird 2023) and calculated the proportion of observations that were heard or seen according to each type of comment.

### 2.4 Patterns in Vocalization Phenology

We extracted mean monthly precipitation at the location of each record, for the month of observation, using WorldClim v2 (Fick and Hijmans 2017), an interpolated monthly climate data set, averaged over 1970 to 2000, and produced at a spatial resolution of 30 s (~1 km^2^). Such precipitation patterns over our broad study area remain relatively stable over time.

To be able to group records within similar precipitation regimes, we clustered monthly precipitation (from the same data source as above) across Central and South America into five broad precipitation regimes, using k‐means clustering, broadly following methods in Ferreira and Reboita (2022) using the R stats package (R Core Team 2023). First, we sampled monthly precipitation at 30,000 random locations, performed k‐means clustering, and then projected the cluster membership across the gridded precipitation data set, aggregated at a 5 km^2^ resolution. We explored silhouette plots, cluster sizes and the ratio of between‐ to total‐sum‐of‐squares to assess clustering performance and inform the number of groups. However, we note that practical considerations for the subsequent modeling (e.g., having sufficient observations per group) was also considered in choosing the number of clusters (Ferreira and Reboita 2022). We added the precipitation regime of the location of each record to the data set (Figure 1a). For locations outside the extent of the precipitation raster, that is, near the coast (*n* = 1484), we assigned the nearest precipitation regime to those records within 5 km and removed the rest (*n* = 89). These spatial mismatches are most likely due to loss of resolution in the rasterization process rather than location errors.

To create the response variable, we summed the number of species records per month, per 15° latitudinal band (from −30° to 30°; Figure 1b) and per precipitation regime. Given the assumption that most records are vocalizations and therefore the difficulty in establishing how many individuals are calling, we treated each observation as a single detection in the summed species records above. A total of 466 records fell below 30° S and were removed to mirror the northern latitude bands. Additionally, these regions would cover non‐Tropical regions. For each of these summarized observations, we calculated daylength (sunrise to sunset) for a representative date (15th) of each month at the centroid of each group of locations, using the R package suncalc (Thieurmel and Elmarhraoui 2022).

### 2.5 Statistical Analysis

To evaluate the effect of daylength and precipitation on vocalization activity in forest tinamous, we performed a generalized additive model (GAM) using the *mgcv* package in R with a negative binomial distribution (Wood 2017). For the response (vocalization activity) we used the total count of tinamou records (as described above), with daylength and mean precipitation as fixed effect smooth terms with thin plate splines; month with a cyclic smooth; and species, latitudinal band, and precipitation regime were included as random effect smooths, using the same representation as smooths in the model, that is, as penalized regression terms (Wood 2017).

To assess the effect of daylength and precipitation within individual latitudinal bands and precipitation regimes, respectively, we ran the model again but included a replicate of each fixed term smooth for each level of latitudinal band and precipitation regime; and therefore, also included each factor as a main effect (Wood 2017). To visualize individual covariate effects on the response, we created “partial effects or response plots,” where the model is predicted along a covariate gradient (within the range of covariate values used in the model) with all other covariates held at their mean value. All code, including the model formulae, is available in the Supporting Information (see below).

## 3 Results

After filtering, we obtained a total of 44,071 records of 16 forest tinamou species from eBird observations between January 2000 and December 2022, with a median number of 1828 records per species (min: 306; Brazilian tinamou *Crypturellus strigolusos*, max: 12,326; Little tinamou *Crypturellus soui* ; Table S2a). Total records across each latitudinal band ranged from 7906 (−15° N to −30° N) to 17,847 (15° N to 0° N; Table S2b). Across precipitation regimes (defined in Figure 1), records ranged from 1785 within precipitation regime 3 to 17,163 in precipitation regime 1 (Table S2c).

### 3.1 Validation of eBird Data

**FIGURE 2:**
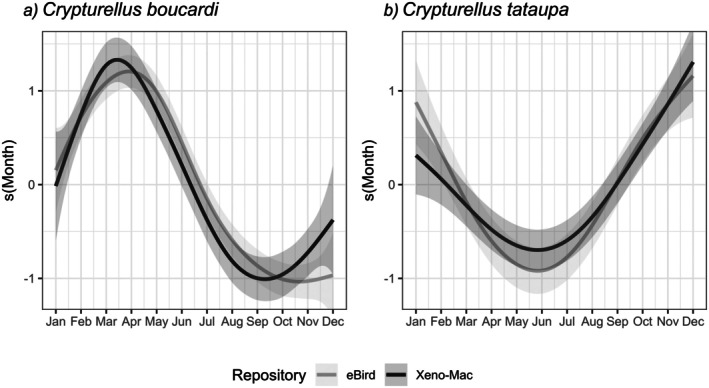
Annual vocal phenology for two tinamous species, (a) *C. tataupa* and (b) *C. boucardi* , modeled using GAMs from both eBird data and combined audio repositories (xeno‐canto and Macaulay Library), shows that both platforms capture the same fundamental signal. This close relationship validates the use of eBird data for studies of vocal phenology at broader regional scales.

Our analysis demonstrates that eBird data captures the same fundamental vocal phenological signal as data from dedicated audio repositories (Figure 2). Our analysis is based on 1453 records of *C. boucardi* (1255 on eBird, 64 on xeno‐canto and 134 on Macaulay), and 3060 records of *C. tataupa* (2768 on eBird, 262 on xeno‐canto and 30 on Macaulay). Cross‐correlation of the GAM‐fitted curves revealed a near‐perfect correlation of 0.98 ( *C. boucardi* ) and 0.97 ( *C. tataupa* ) between the datasets after a minor temporal adjustment. This high degree of congruence indicates virtually identical seasonal patterns in vocal activity. The need for a small phase shift (approximately 0.45 months for *C. boucardi* and 0.0 months for *C. tataupa* ) to achieve this alignment indicates a consistent, predictable difference in the precise timing of peak detection between the platforms but does not detract from the overall similarity of the phenological shapes they record.

In terms of annotations on the eBird records, of a total of 6273 records with any comments or behavior codes, only 2% corresponded to sight records whereas 49% corresponded to heard records. The remaining comments were on aspects other than vocalizations. Observations corresponding to vocalizations included “singing male” in the behavior code and the Spanish or English words, for example, “heard” or “escucha” in the comments. Comments implying sight records included the terms “seen,” “visto,” or “vimos” (Table S1). The authors have extensive field experience and can corroborate the relative frequency of seen versus heard records for Tinamidae species.

### 3.2 Patterns in Vocalization Phenology

**FIGURE 3:**
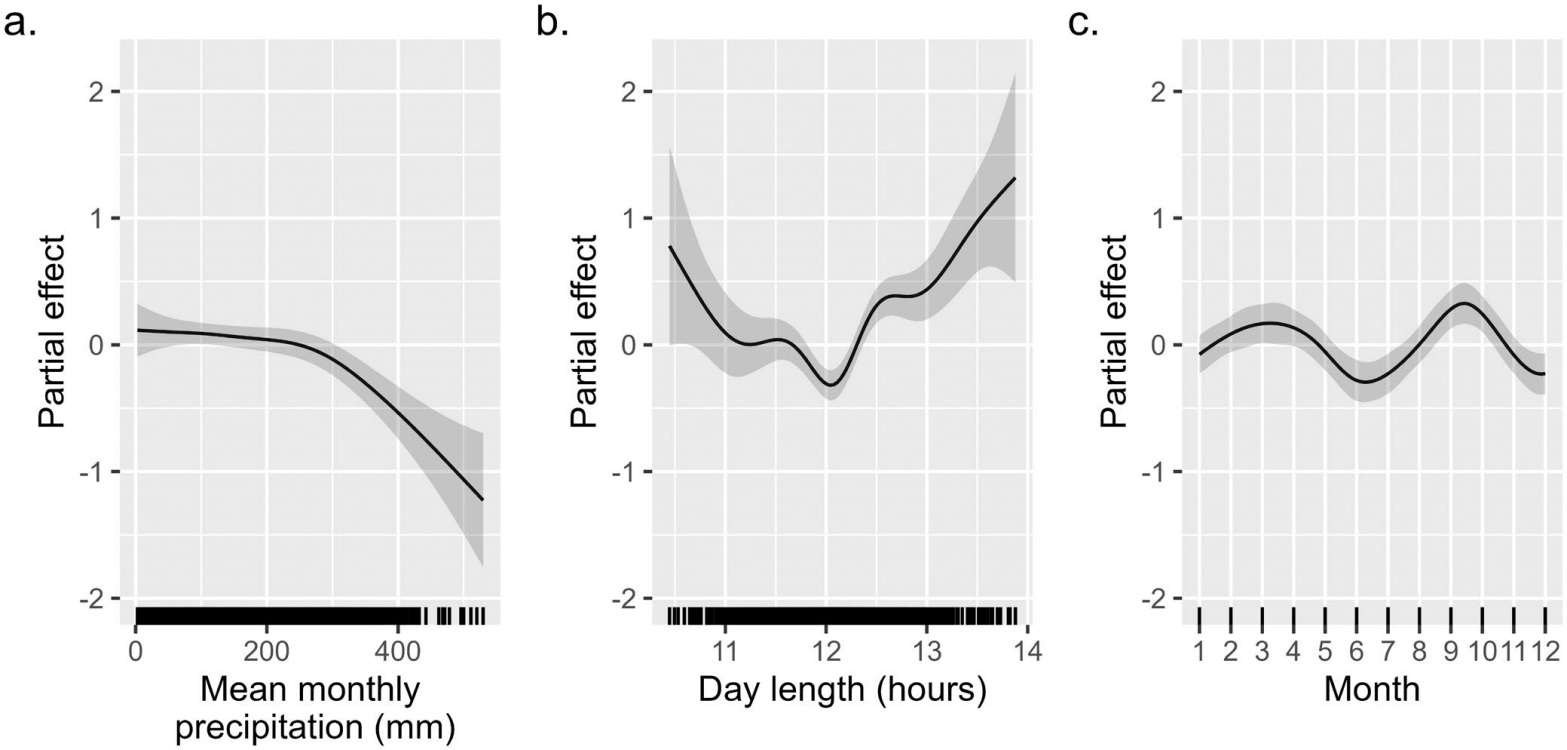
Partial response curves from generalized additive models: Number of tinamou vocalization records (a) decrease with increasing mean monthly precipitation; (b), generally increase with increasing daylength; (c) peak bimodally around March–April and September–October. Note that months run from January (1) to December (12).

We found two general patterns in the phenology of vocalizations that were strongly associated with changes in precipitation and changes in daylight hours across all latitudinal bands and months (Figure 3, Table S3). Vocalizations tend to decrease with increasing precipitation (Figure 3a) and increase with increasing daylengths (hours between sunrise and sunset; Figure 3b), with two peaks per year between March–April and September–October (Figure 3c). The model had an explained deviance of 57.6%.

**FIGURE 4:**
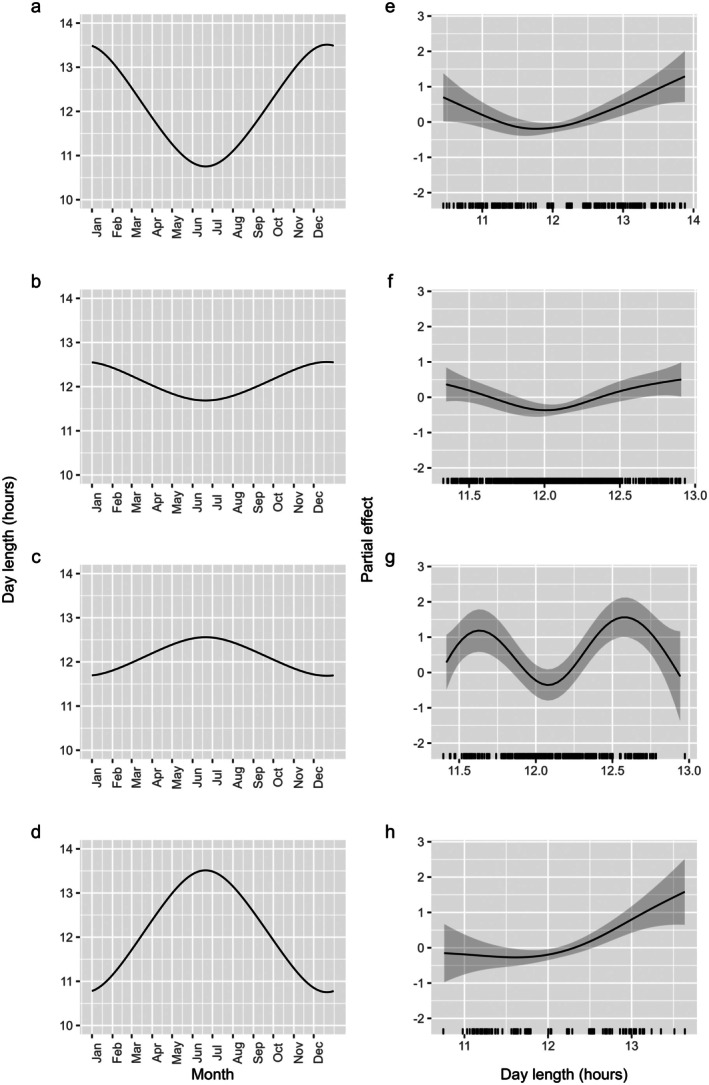
Partial response curves from generalized additive models by latitude band (from north to south; e–h). The left panels (a–e) illustrate change in daylength (dawn to dusk) over four latitudinal bands (a: 30° N to 15° N, b: 15° N to 0°, c: 0° to 15° S, d: 15° S to 30° S) for the mean longitude of records within the band. The effect of daylength on the number of tinamou vocalizations per month is more consistent and stronger at higher (e) and lower (h) latitudinal bands than those nearer the Equator (f, g).

**FIGURE 5:**
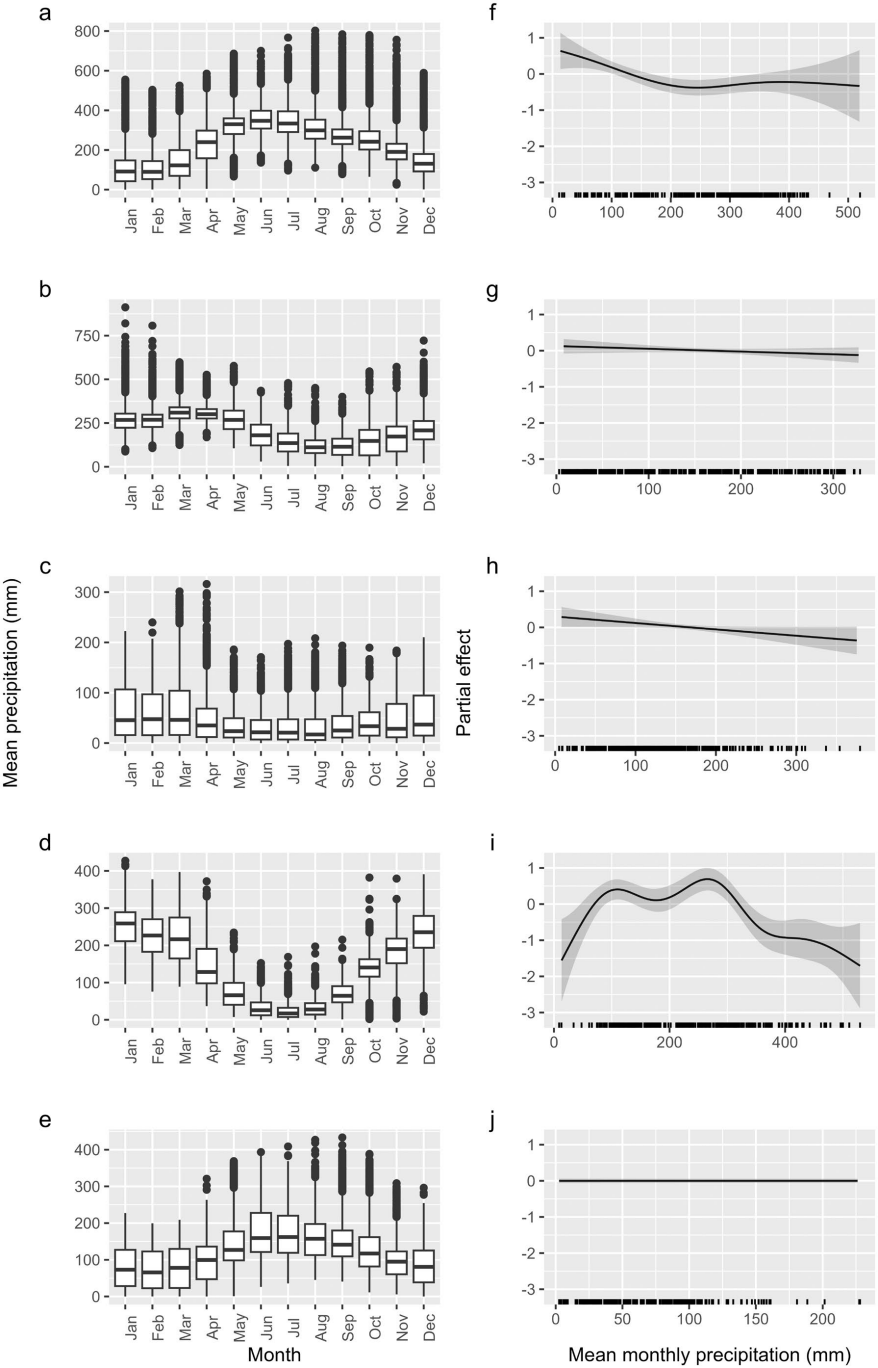
Partial response curves from generalized additive models by latitude band. The effect of mean monthly precipitation on number of tinamou vocalizations per month is stronger within northern South America/Central America (f) and northern Amazonia (i); also the regions with most rainfall per year. Other regions (g, h) show less effect of precipitation on number of vocalizations per month or none (j). The left panels (a–e) show representative annual precipitation patterns for the five regimes (see Figure 1).

When the smooth terms were allowed to vary by latitudinal band (for effect of daylength) and by precipitation regime (for effect of mean annual precipitation), deviance explained dropped to 48%. A positive effect of daylength on tinamou vocalizations was strongest furthest from the equator (Figure 4e,h; Table S4). This aligns with the fact that the variation in daylength amplitude increases with absolute latitude, although either side of the equator, the effect is the same, in that calls increase with increasing daylength beyond 12 h. However, the effect of rainfall on vocalizations was less clear (Figure 5; Table S3), with significant effects for three precipitation regimes, in northern South America/Central America (precipitation regime 1, 3; see Figure 1) and northern Amazonia (regime 4). However, the effect size is larger for those regions with larger variation in precipitation (Figure 5).

## 4 Discussion

The strong correlation (*r* > 0.95) between eBird and audio repository phenologies provides robust validation for using broad‐scale citizen science data in vocal phenology research. This finding confirms that the seasonal patterns of avian vocal activity derived from incidental birdwatcher observations are consistent with those derived from targeted acoustic recordings for species that are predominantly heard more than seen. The minimal phase shift required for optimal alignment likely reflects predictable, platform‐specific characteristics—such as differences in observer effort—rather than a discrepancy in the underlying biological signal. Consequently, our results strongly support eBird as a reliable and powerful resource for studying vocal phenology at regional and continental scales.

Vocal activity studies in tropical areas increased in the last decade (Berman et al. 2024; Quispe et al. 2017; Goymann et al. 2012), yet most of these studies focus on single species in a particular area. These studies have shown that most birds exhibit seasonal phenology adapted to local changes in climatic conditions (Hau et al. 1998; Wikelski et al. 2000, 2003). In contrast, vocal activity of temperate zone birds is highly predictable and seasonal with a clear peak in the spring before the nesting season (Stutchbury and Morton 2022). Although most tropical birds nest at the start of the rainy season, nesting shows greater variability within and among species (Stouffer et al. 2013). This might explain why phenological patterns in the tropics are often less obvious than in temperate regions.

In this study, we found evidence for seasonality in the vocal phenology (corroborated as the number of eBird detections) of 16 tinamou species across their whole ranges, linked to changes in daylength and precipitation patterns. Similar patterns have been observed in other tinamous, such as *Crypturellus boucardi* in Honduras and *Crypturellus noctivagus* in the Brazilian Pantanal (Lancaster 1964; Pérez‐Granados and Schuchmann 2021).

Within the tropics, two phenological strategies have been described: some species breed seasonally, while others breed unseasonally (Stutchbury and Morton 2022). For example, Berman et al. (2024) documented a marked difference in the breeding phenology of forest birds versus parkland colonizers in Singapore. Native species, which are adapted to forest interiors, exhibited seasonal vocalizations, while species that had established populations in open habitats within the last few decades vocalized throughout the year (Berman et al. 2024). In our study, species that inhabit dense forest interiors exhibited a marked vocal phenological period that varied with latitude. The vocal period increased with daylength and decreased with rainfall throughout the year (see Figure 3).

In temperate regions, temperature fluctuations influence food abundance, which in turn affects the timing of breeding (Thompson and Willson 1979). In contrast, near the equator, temperature is more stable, and precipitation becomes the primary driver of food availability and breeding (Hau et al. 2008). Food abundance for insectivorous and many frugivorous birds increases dramatically during the early rainy season (young leaves are palatable for many arthropods, determining their phenology), which marks the onset of their breeding season (Hau et al. 2008).

Our results indicate that vocalizations in tinamous decrease as mean monthly precipitation increases, suggesting that courtship displays occur at the end of the dry season just before the beginning of the rainy season. For instance, the breeding season of *C. boucardi* coincides with the dry season in Belize, which extends from January to May (Lancaster 1964). In contrast, the peak of the breeding season for *C. soui* in southwestern Colombia occurs during the rainy season, between September and December (Corredor‐Londoño et al. 2022).

Our data showed bimodal peaks in vocal (and breeding) activity, with peaks in March–April and September–October, corresponding to increasing daylength in the southern and northern hemispheres, respectively (see Figure 1a–d). Because birds in opposite hemispheres respond to reciprocal changes in daylength, their breeding seasons are temporally offset (e.g., northern summer vs. southern summer). This hemispheric asynchrony means that breeding activity occurs somewhere in the tropics throughout the year, giving rise to the aggregate pattern of an extended tropical breeding season. A similar pattern is observed in tropical plants, where two annual maxima of advancing sunset/sunrise times at the equinoxes correspond to two annual flowering periods (Rivera et al. 2002; Borchert et al. 2005). Plants that flower before the autumn equinox are considered “short‐day plants”, because they respond to the contraction of the day, whereas others are considered “long‐day plants” because they flower before the spring equinox, when the days are getting longer (Rivera et al. 2002; Borchert et al. 2005).

Our results, which demonstrate a seasonal pattern in tinamou vocal activity, align with the hypothesis that tropical birds can use minimal photoperiodic change as a predictive cue for breeding. A mechanistic link may be the Intertropical Convergence Zone (ITCZ), whose seasonal migration is the ultimate driver of tropical rains and is itself correlated with hemispheric differences in solar insolation and daylength (Waliser and Gautier 1993). Even at equatorial latitudes, the consistent, albeit small, increase in daylength could provide a reliable signal that precedes the ITCZ‐driven onset of precipitation. For visually cryptic tinamous in the dense understory, this astronomical cue may be more reliable than variable local weather, allowing them to initiate breeding behaviors in anticipation of optimal resource conditions.

The study of vocal phenology has grown significantly in recent years due to advances in autonomous recording technology. However, other sources of information, such as online citizen science repositories, including eBird (eBird 2023) and xeno‐canto (www.xeno‐canto.org), may prove to be useful proxies for studying vocal phenology at larger continental scales (Rueda‐Uribe et al. 2024; Laverde‐R et al. 2017), as demonstrated in this study. This approach may be especially beneficial for studying forest birds that are difficult to see but easy to hear, such as tinamous, antpittas, tapaculos, and some thrushes, among others.

Recently, tropical phenology, especially that of plants, has gained attention within the scientific community as we attempt to explain and understand the consequences of ecosystem changes taking place in response to a changing climate (Abernethy et al. 2018). Similarly, song phenology may also serve as a useful biological indicator of climatic variation within and among regions, which could be used to measure the impact of human‐induced climate change on bird behavior (Bates et al. 2023) and inform large‐scale conservation efforts in the region (Devenish et al. 2009). A collaborative effort between singing birds, birdwatchers, and scientists could be part of a comprehensive strategy to monitor and address the changes and challenges facing the planet today.

## Author Contributions

**Oscar Laverde‐R.:** conceptualization (equal), funding acquisition (equal), investigation (equal), methodology (equal), visualization (equal), writing – original draft (equal), writing – review and editing (equal). **Santiago Muñoz Bolaños:** conceptualization (equal), data curation (equal), investigation (equal), methodology (equal), writing – original draft (equal), writing – review and editing (equal). **Gustavo A. Londoño:** conceptualization (equal), investigation (equal), methodology (equal), visualization (equal), writing – original draft (equal), writing – review and editing (equal). **Christian Devenish:** conceptualization (equal), data curation (equal), formal analysis (equal), investigation (equal), methodology (equal), visualization (equal), writing – original draft (equal), writing – review and editing (equal).

## Funding

O.L.‐R. was funded by grant no. 20642 from the Vicerrectoría de Investigaciónes of the Pontificia Universidad Javeriana, Bogotá.

## Ethics Statement

No data was specifically collected for this project. eBird encourages ethical birding among those who submit records by following its community guidelines (https://support.ebird.org/en/support/solutions/articles/48001257989‐ebird‐and‐merlin‐community‐guidelines).

## Conflicts of Interest

The authors declare no conflicts of interest.

## Supporting information


**Table S1:** Number of observation annotations (*n* = 6273) with evidence of record type (sight or vocalization).
**Table S2a:** Number of records per species of Tinaminae clade after filtering (see methods) obtained from eBird observations between January 2000 and December 2022.
**Table S2b:** Number of records per latitudinal band.
**Table S2c:** Number of records per precipitation regime.
**Table S3:** Generalized additive model—summary of smooth terms. See methods for details of the model data.
**Table S4:** Generalized additive model—summary of smooth terms for model including separate smooths for each latitudinal band and precipitation regime.

## Data Availability

eBird data is available on request from https://ebird.org/data/download; climate data is freely available from https://www.worldclim.org/ for academic research. Analysis code for this project is available at https://github.com/Cdevenish/tinamidae_song_phenology.
