# Supplementary material for: Vocal Phenology of Forest Tinamous Along a Latitudinal Gradient: Effects of Daylength and Precipitation
Source: Ecol Evol. 2026 Jun 12;16(6):e73741. doi: 10.1002/ece3.73741 (PMC13261373; doi:10.1002/ece3.73741)
Supplement: Supplementary file 1 — Table S1: Number of observation annotations (n = 6273) with evidence of record type (sight or vocalization). Table S2a: Number of records per species of Tinaminae clade after filtering (see methods) obtained from eBird observations between January 2000 and December 2022. Table S2b: Number of records per latitudinal band. Table S2c: Number of records per precipitation regime. Table S3: Generalized additive model—summary of smooth terms. See methods for details of the model data. Table S4: Generalized additive model—summary of smooth terms for model including separate smooths for each latitudinal band and precipitation regime. [file ECE3-16-e73741-s001.docx]

**Supplementary Info**

**Table S1.** Number of observation annotations (n = 6,273) with evidence of record type (sight or vocalisation).

| **Annotation type** | **Total records with annotations** | **Implied sight record** | **Implied vocalisation** |
| --- | --- | --- | --- |
| **Behaviour code** | 1,199 | 30  (e.g. feeding young, nest with eggs) | 940  (e.g. singing male) |
| **Comment** | 5,480 | 111  (e.g. “seen”, “visto” “vimos”) | 2,163  (e.g. “heard”, “eschucha”) |

**Table S2a.** Number of records per species of Tinaminae clade after filtering (see methods) obtained from eBird observations between January 2000 and December 2022.

| **Name** | **Scientific Name** | **No. of records** |
| --- | --- | --- |
| Little Tinamou | *Crypturellus soui* | 12,326 |
| Great Tinamou | *Tinamus major* | 8,434 |
| Thicket Tinamou | *Crypturellus cinnamomeus* | 4,813 |
| Undulated Tinamou | *Crypturellus undulatus* | 4,278 |
| Small-billed Tinamou | *Crypturellus parvirostris* | 2,963 |
| Brown Tinamou | *Crypturellus obsoletus* | 2,476 |
| Cinereous Tinamou | *Crypturellus cinereus* | 2,409 |
| Tataupa Tinamou | *Crypturellus tataupa* | 2,286 |
| Slaty-breasted Tinamou | *Crypturellus boucardi* | 1,245 |
| Variegated Tinamou | *Crypturellus variegatus* | 484 |
| Grey Tinamou | *Tinamus tao* | 458 |
| Solitary Tinamou | *Tinamus solitarius* | 457 |
| Highland Tinamou | *Nothocercus bonapartei* | 412 |
| Pale-browed Tinamou | *Crypturellus transfasciatus* | 366 |
| White-throated Tinamou | *Tinamus guttatus* | 358 |
| Brazilian Tinamou | *Crypturellus strigulosus* | 306 |

**Table S2b.** Number of records per latitudinal band

| **Latitudinal band** | **No. of records** |
| --- | --- |
| -30 to -15 | 7,906 |
| -15 to 0 | 8,789 |
| 0 to 15 | 17,847 |
| 15 to 30 | 9,529 |

**Table S2c.** Number of records per precipitation regime (see Figure 1a; Figure 5a-e, corresponding to regimes 1-5, respectively)

| **Precipitation regime** | **No. of records** |
| --- | --- |
| 1 | 17,163 |
| 2 | 3,045 |
| 3 | 1,785 |
| 4 | 10,175 |
| 5 | 11,903 |

**Table S3.** Generalised additive model – summary of smooth terms. See methods for details of the model data.

| **Covariate** | **edf** | **Reference degrees of freedom** | **Chi square** | **p-value** |
| --- | --- | --- | --- | --- |
| s(mean_prec) | 2.90 | 9 | 1074.71 | < 0.001 |
| s(day_length) | 6.73 | 9 | 2149.22 | < 0.001 |
| s(month_no) | 5.94 | 10 | 54.08 | < 0.001 |
| s(scientific_name) | 14.49 | 15 | 2435.31 | < 0.001 |
| s(k5,latBand) | 16.65 | 17 | 5055.59 | < 0.001 |

**Table S4.** Generalized additive model – summary of smooth terms for precipitation and day length by precipitation regime and latitudinal band, respectively

| **Covariate** | **edf** | **Reference degrees of freedom** | **Chi square** | **p-value** |
| --- | --- | --- | --- | --- |
| s(mean_prec):k51 | 2.632 | 9 | 17.136 | <0.001 |
| s(mean_prec):k52 | 0.62 | 9 | 1.562 | 0.125 |
| s(mean_prec):k53 | 0.81 | 9 | 4.208 | 0.026 |
| s(mean_prec):k54 | 6.155 | 9 | 70.359 | <0.001 |
| s(mean_prec):k55 | 0.001 | 8 | 0.001 | 0.406 |
| s(day_length):latBand-30 to -15 | 2.832 | 9 | 38.323 | <0.001 |
| s(day_length):latBand-15 to 0 | 3.16 | 9 | 46.031 | <0.001 |
| s(day_length):latBand0 to 15 | 5.29 | 9 | 164.589 | <0.001 |
| s(day_length):latBand15 to 30 | 2.115 | 9 | 21.203 | <0.001 |
| s(month_no) | 6.335 | 10 | 65.688 | <0.001 |
| s(scientific_name) | 14.533 | 15 | 525.921 | <0.001 |
